# Combined Inhibition of the TGF-β1/Smad Pathway by *Prevotella copri* and *Lactobacillus murinus* to Reduce Inflammation and Fibrosis in Primary Sclerosing Cholangitis

**DOI:** 10.3390/ijms241311010

**Published:** 2023-07-02

**Authors:** Yu Shen, Baorong Jiang, Chenchen Zhang, Qian Wu, Lei Li, Ping Jiang

**Affiliations:** 1 Center for Global Health, School of Public Health, Nanjing Medical University, 101 Longmian Avenue, Nanjing 211166, China; 2 Key Lab of Modern Toxicology of Ministry of Education, School of Public Health, Nanjing Medical University, 101 Longmian Avenue, Nanjing 211166, China

**Keywords:** *Prevotella copri*, *Lactobacillus murinus*, PSC, inflammation, fibrosis, TGF-β1/Smad pathway

## Abstract

Primary sclerosing cholangitis (PSC) is a chronic cholestatic disease characterized by inflammation and fibrosis of the bile ducts. Cholestasis may lead to hepatic inflammation and fibrosis, and amelioration of cholestasis may allow recovery from inflammatory and fibrotic pathological damage. *Prevotella copri* (*P. copri*) interventions have been reported to significantly improve cholestasis and liver fibrosis in 3,5-diethoxycarbonyl-1,4-dihydrocollidine (DDC)-induced PSC mouse models. Even though *P. copri* treatment alone cannot bring about recovery from DDC-induced inflammation, it increases the abundance of *Lactobacillus murinus* (*L. murinus*) compared with DDC treatment, which has been reported to have anti-inflammatory effects. The abundance of L. murinus still not recovering to a normal level may underlie hepatic inflammation in *P. copri* + DDC mice. Separate or combined interventions of *P. copri* and *L. murinus* were used to investigate the molecular mechanism underlying the improvement in PSC inflammation and fibrosis. *P. copri* and *L. murinus* significantly reduced the hepatic inflammatory cell aggregation and inflammatory factor expression as well as the hepatic collagen content and fibrin factor expression in the PSC mice. Further analysis of phosphorylation and dephosphorylation levels revealed that treating the PSC mice with the *P. copri* and *L. murinus* combined intervention inhibited the activity of the DDC-activated TGF-β1/Smad pathway, thereby reducing liver inflammation and fibrosis. The combination of *P. copri* and *L. murinus* inhibits the TGF-β1/Smad pathway and reduces inflammation and fibrosis in PSC.

## 1. Introduction

Primary sclerosing cholangitis (PSC) is a chronic cholestatic liver disease of unclear etiology [1,2,3]. With long-term cholestasis and inflammatory and fibrotic lesions, most patients eventually develop cirrhosis and liver failure with poor prognosis [4,5]. There are no effective drugs available, which stimulates the need for research to elucidate interventions and treatments for PSC. Studies have confirmed that the intestinal microbiota of the PSC population is disturbed and *P. copri* is significantly deficient in PSC patients [6,7,8]. In our previous study, we constructed a PSC mouse model exhibiting bile duct stricture, liver inflammation, and fibrosis using a 0.1% (*w*/*w*) 3,5-diethoxycarbonyl-1,4-dihydrocollidine (DDC)-supplemented diet [9]. Subsequently, *P. copri* was administered to the PSC model animals to further explore the effect of the microbial intervention. Our previous study found that the symptoms of cholestasis of the PSC mice significantly improved after the *P. copri* treatment, but *P. copri* did not reduce liver inflammation [9]. 16S rRNA sequencing analysis determined that *P. copri* could regulate the microenvironment of intestinal microbiota by changing the abundance of specific genera, among which the abundance of *Lactobacillus* genus, the top-ranked beneficial bacteria, increased after *P. copri* intervention in PSC mice, with the most abundant species being *L. murinus*. In addition, studies have found that *L. murinus* could mediate anti-inflammatory effects in calorie-restricted mice and has a significant effect in reducing inflammation [10,11,12]. Therefore, in this study, we hypothesized that *L. murinus* may assist *P. copri* in improving inflammation in the DDC group.

Chronic inflammation and fibrosis manifested by PSC is a compensatory response to tissue repair following cholestatic injury secondary to intra- and extrahepatic biliary stenosis [13]. Important fibrogenic cytokines, such as transforming growth factor β1 (TGF-β1), are largely activated and released in injured bile duct and liver tissue [14,15]. TGF-β1 plays an important role in hepatic stellate cell (HSC) activation and liver tissue fibrosis by sequentially binding to its receptors TGF-βR II and TGF-βR I, thereby regulating the expression of drosophila mothers against decapentaplegic protein (SMAD) and its phosphorylated proteins to complete the nuclear translocation of TGF-β1/Smad signaling [16,17,18,19]. In this study, we used a combined intervention of *P. copri* and *L. murinus* in PSC mice and found that *P. copri* and *L. murinus* alone or in combination improved the serological indices of hepatic cholestasis and the degree of liver fibrosis in PSC mice. In particular, *L. murinus* exerted its specific role in reducing liver inflammation by decreasing the hepatic infiltration of macrophages and monocytes in the PSC mice, and this inflammation-mitigating effect was promoted when co-intervened with *P. copri*. Further analysis revealed that the additive bacterial intervention inhibited the DDC-activated TGF-β1/Smad pathway and significantly reduced total SMAD2/3 proteins and phosphorylated proteins, which suggested that *P. copri* and *L. murinus* alleviated liver inflammation and fibrosis in the PSC mice by inhibiting TGF-β1/Smad signaling and transduction. Collectively, these results suggest the potential of combined microbial intervention of *P. copri* and *L. murinus* as a therapy for treating inflammation and fibrosis in PSC.

## 2. Results

### 2.1. P. copri and L. murinus Coordinately Improve DDC-Induced Liver Damage, Bile Duct Obstruction, and Cholestasis

**Figure 1:**
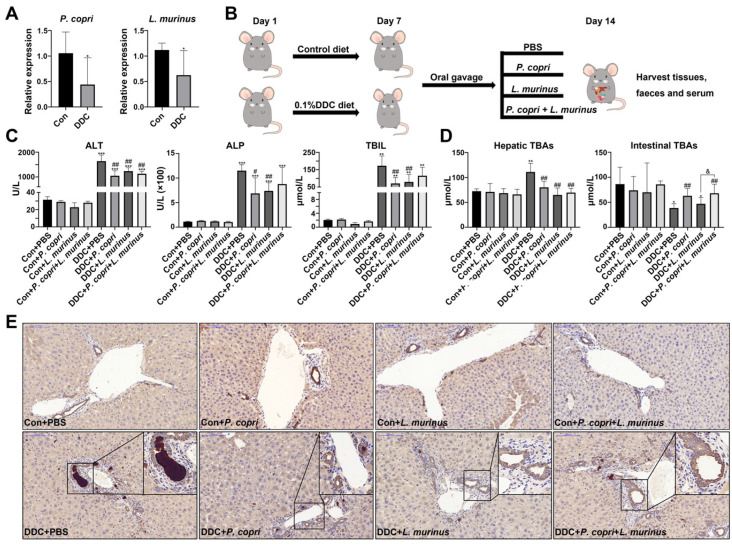
Supplementation of *P. copri* and *L. murinus* improves liver injury and cholestasis in PSC mice. (**A**) The abundance of *P. copri* and *L. murinus* in PSC mice. (**B**) Schematic diagram of PSC mouse construction and intervention method in this experiment. (**C**) The levels of ALT, ALP, and TBIL in the serum of mice in each group. (**D**) TBA content in liver and small intestinal tissues. (**E**) Immunohistochemistry staining of CK19 in liver tissues, and the box shows the obstruction of bile components in the bile duct. (Scale bar = 100 μm; original magnification 20×; * indicates statistical difference compared to the Con + PBS (Con is short for control) group, # indicates statistical difference compared to the DDC + PBS group, and & indicates statistical difference compared to the DDC+ *P. copri* + *L. murinus* group; * *p* < 0.05, ** *p* < 0.01, *** *p* < 0.001; # *p* < 0.05, ## *p* < 0.01; and ^&^ *p* < 0.05).

Our previous study confirmed that the *Lactobacillus* genus was decreased in DDC-induced PSC mice and monocolonized Gram-negative *P. copri* could recover its abundance, and further analysis found that *Lactobacillus murinus* (*L. murinus*) was the most abundant Gram-positive bacterium in the DDC group [9] (Figure S1A,B). The qPCR analysis revealed a noticeable decrease in the abundance of *P. copri* and *L. murinus* within the DDC group when compared with the control group, confirming our previous research findings (Figure 1A). After that, an experimental schematic diagram was designed, as shown in Figure 1B; in the experiment, the control and PSC model mice were fed a normal diet or the 0.1% (*w*/*w*) DDC diet for one week, respectively. Subsequently, the mice were orally administered bacterial suspensions of *P. copri* and *L. murinus*, either alone or in combination for an additional week. The serum levels of ALT, ALP, and TBIL were significantly elevated in the DDC group compared with the control mice, indicating that DDC induced liver damage, bile duct obstruction, and cholestasis (Figure 1C). Compared with the DDC group, administering *P. copri* or *L. murinus* alone significantly reduced the levels of ALT, ALP, and TBIL in the DDC-induced groups (Figure 1C), while administering *P. copri* and *L. murinus* together significantly reduced the level of ALT in the DDC-induced groups (Figure 1C). There was no significant difference between the groups receiving *P. copri* or *L. murinus* treatment alone and the combined treatment group. Notably, the intervention of *P. copri* and *L. murinus* in the PSC mice resulted in a reduction in hepatic TBAs to a level comparable to that of the control mice (Figure 1D). Similarly, the *P. copri* or *P. copri + L. murinus* treatment blunted the reducing effect on intestinal TBAs in the DDC-induced mice, except for the *L. murinus* treatment (Figure 1D). Furthermore, the high expression of the bile duct epithelial marker cytokeratin 19 (CK19) after being fed the DDC diet was attenuated by the *P. copri* and/or *L. murinus* treatment, which further confirmed that *P. copri* and/or *L. murinus* intervention could alleviate the obstruction of the bile duct induced by the DDC diet in liver (Figure 1E).

### 2.2. Supplementation with P. copri Accelerates the Improvement That L. murinus Has on Inflammation in PSC Mice

**Figure 2:**
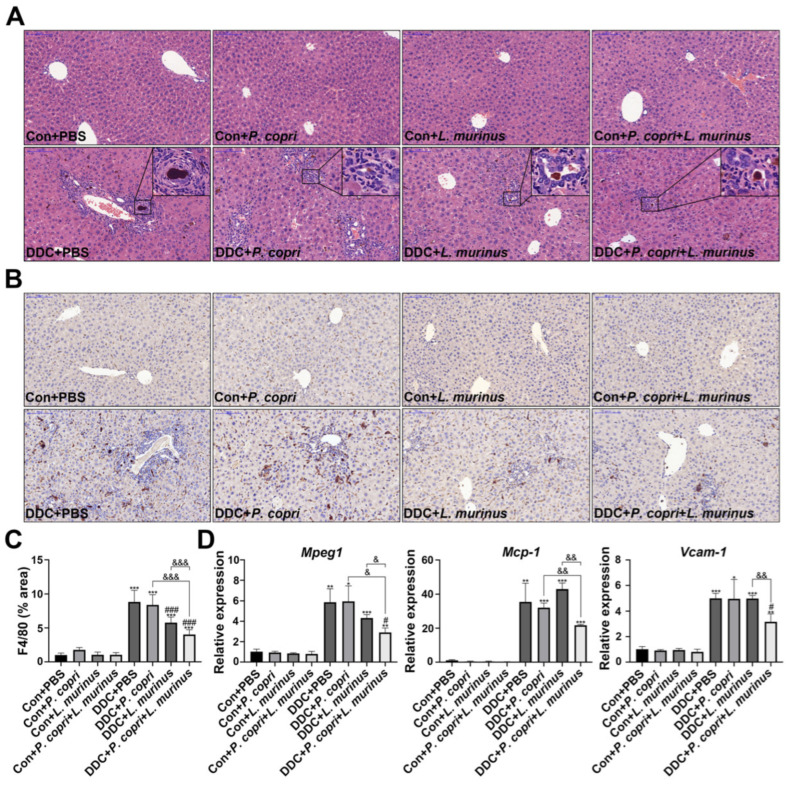
*L. murinus* combined with *P. copri* significantly reduces liver inflammation in PSC mice. (**A**) H&E staining of liver tissues reveals the aggregation of inflammatory cells, as shown in the box. (**B**) The changes in macrophages in liver tissues were observed using F4/80 immunohistochemical staining. (**C**) The area of F4/80 positive staining in liver tissues was quantitatively determined using ImageJ software (version 2.3.0), and six fields were randomly selected from the staining map of each sample section in each group for analysis. (**D**) The secretion levels of *Mpeg1*, *Mcp-1,* and *Vcam-1* in liver tissues. (Scale bar = 100 μm; original magnification 20×; * indicates statistical difference compared to the Con + PBS group, # indicates statistical difference compared to the DDC + PBS group, and & indicates statistical difference compared to the DDC + *P. copri* + *L. murinus* group; * *p* < 0.05, ** *p* < 0.01, *** *p* < 0.001; # *p* < 0.05, ### *p* < 0.001; ^&^ *p* < 0.05, ^&&^ *p* < 0.01, and ^&&&^ *p* < 0.001).

The H&E staining showed that the DDC treatment induced the aggregation of a large number of inflammatory cells in the liver to activate inflammatory response, while the addition of *P. copri* and *L. murinus* intervention resulted in a noticeable reduction in both the extent and number of inflammatory cell infiltrates (Figure 2A). IHC staining, along with quantitative analysis, was employed to assess the expression of the macrophage marker F4/80 in the liver tissues of the mice (Figure 2B,C). According to the quantitative results (Figure 2C), compared with the intervention with *P. copri* or *L. murinus* alone, the combined intervention can significantly reduce the expression of F4/80, indicating that the combined intervention of the two strains has a better effect on reducing liver inflammation caused by the DDC diet than the single bacterial intervention. The above finding was corroborated by reevaluating the mRNA levels of the macrophage marker *Mpeg1* (Figure 2D). Furthermore, the suppression of monocyte chemoattractant protein-1 (*Mcp-1*) and vascular cell adhesion factor (*Vcam-1*) is responsible for the effect of the combined intervention of *L. murinus* and *P. copri*, which indicates that the combined intervention with these two bacteria might reduce the inflammation induced by the DDC diet through reducing the migration and infiltration of macrophages (Figure 2D). However, the intervention with *P. copri* or *L. murinus* alone did not decrease the expression of *Mcp*-1 and *Vcam-1,* further confirming that the *P. copri* treatment facilitates the improvement that *L. murinus* has on inflammation in PSC mice.

### 2.3. Coordination between P. copri and L. murinus Downregulates DDC-Induced Liver Fibrosis

**Figure 3:**
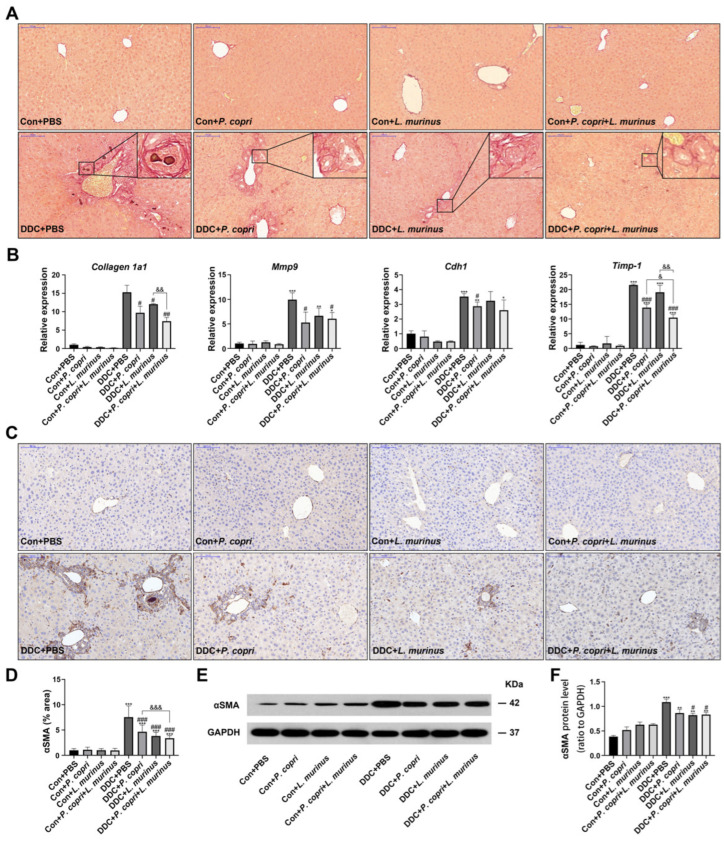
*P. copri* and *L. murinus* alone and in combination can improve liver fibrosis in PSC mice. (**A**) SR staining of liver tissues from mice, with onion-like fibrotic phenotype shown in the box. (**B**) Expression levels of fibrosis-related factors *Cdh1*, *Collagen 1a1*, *Timp-1,* and *Mmp9* in liver tissues. (**C**) αSMA immunohistochemical staining of liver tissues. (**D**) Statistical analysis of positive staining area analyzed using ImageJ software. (**E**) The bands of αSMA protein and internal reference GAPDH protein in liver tissues of each group of mice. (**F**) The expression level of αSMA protein relative to GAPDH protein. (Scale bar = 100 μm; original magnification 20×; * indicates statistical difference compared to the Con + PBS group, # indicates statistical difference compared to the DDC + PBS group, and & indicates statistical difference compared to the DDC + *P. copri* + *L. murinus* group; * *p* < 0.05, ** *p* < 0.01, *** *p* < 0.001; # *p* < 0.05, ## *p* < 0.01, ### *p* < 0.001; ^&^ *p* < 0.05, ^&&^ *p* < 0.01, and ^&&&^ *p* < 0.001).

The formation of extracellular matrix causes the aggregation of collagen and its winding spiral arrangement, which drives fibrosis in the liver [20,21]. To further investigate the effect of *P. copri* and/or *L. murinus* on liver fibrosis, SR staining was employed and revealed that the DDC diet could induce onion-like fibrosis by recruiting collagen; either *P. copri* and *L. murinus* alone or in combination induced an apparent alleviation of collagen deposit (Figure 3A). In addition, the qPCR detected that the expression of *Collagen 1a1* in the DDC-induced PSC mice decreased after treatment with *P. copri* (Figure 3A) or *L. murinus* alone, or in combination, which suggests that these interventions may retard type I collagen accumulation in PSC mice. Moreover, we detected the expression of metalloproteinase 9 (*Mmp9*), adhesion molecule E-cadherin/*Cah1*, and tissue inhibitor of metalloproteinase 1 (*Timp-1*) in the liver (Figure 3B). Compared with the control group, the levels of *Mmp9* and *Timp-1* in the DDC group significantly increased, whereas the levels markedly reduced in the DDC + *P. copri* and DDC + *P. copri + L. murinus* groups, suggesting that both interventions significantly slow the accumulation of extracellular matrix and attenuate fibrosis under the background of DDC diet. Nevertheless, *L. murinus* administration could not offset the increase in *Mmp9*, *Cdh1,* and *Timp-1* in the PSC mice, suggesting that the alleviating effect of *L. murinus* treatment alone is not as effective as its co-intervention with *P. copri* in PSC mice. Compared to the DDC + *P. copri* and DDC + *L. murinus* groups, the DDC + *P. copri + L. murinus* group could significantly reduce the expression of the pro-fibrogenic factor *Timp-1* in the PSC mice, indicating that the combined treatment with these two bacteria could better alleviate liver fibrosis caused by the DDC diet. As a marker of HSC activation, α-smooth muscle actin (α-SMA) plays an important role in liver fibrosis. As shown in Figure 3C,D, the IHC analysis demonstrated that the DDC group had the highest α-SMA expression, and both *P. copri* and *L. murinus*, either alone or in combination, significantly decreased the expression of α-SMA in the PSC mice. Consistent with this result, the Western blotting further confirmed that α-SMA protein was also reduced in the PSC mice after being given the treatment with *L. murinus* alone or the combined treatment with the two bacteria (Figure 3E,F). Collectively, these results indicate that administering either *P. copri* or *L. murinus* alone, or both together, could alleviate DDC-induced liver fibrosis by downregulating the expression of *Collagen 1a1, Mmp9*, and α-SMA.

### 2.4. Treatments with P. copri and L. murinus Alone or in Combination Alleviate DDC-Induced Liver Fibrosis by Inhibiting TGF-β1/Smad Signaling

**Figure 4:**
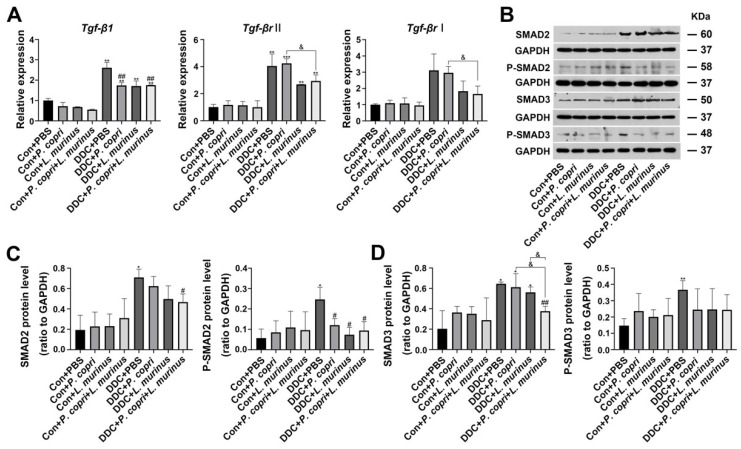
*P. copri* and *L. murinus* reduce liver inflammation and fibrosis in PSC mice by inhibiting the DDC-activated TGF-β1/Smad pathway. (**A**) The expression of TGF-β1 and its receptor, Smad2, in liver tissues was determined using qPCR. (**B**) Immunoblotting bands of total and phosphorylated proteins of SMAD2 and 3. The gray values of the total and phosphorylated protein bands of SMAD2 (**C**) and SMAD3 (**D**) in the liver tissues of mice in each group were statistically analyzed using ImageJ software. (* indicates statistical difference compared to the Con + PBS group, # indicates statistical difference compared to the DDC + PBS group, and & indicates statistical difference compared to the DDC + *P. copri* + *L. murinus* group; * *p* < 0.05, ** *p* < 0.01, *** *p* < 0.001; # *p* < 0.05, ## *p* < 0.01; and ^&^ *p* < 0.05).

To elucidate the underlying mechanism through which *P. copri* or *L. murinus* inhibits fibrosis in the PSC mouse model, we detected the TGF-β1/Smad pathway. First, we found that the DDC diet increased the mRNA expression of *Tgf-β1* and *Tgf-βr II* compared with the control group (Figure 4A). Treatment with *P. copri* alone or combined treatment of *P. copri* and *L. murinus* could offset the increase in *Tgf-β1* observed in the PSC mice. The Western blotting further confirmed that the DDC diet stimulates the release of TGF-β1 to activate the phosphorylation of SMAD2/3, which leads to P-SMAD2/3 translocation from the cytoplasm to the nucleus and induces HSC activation and liver fibrosis (Figure 4B). Compared with the DDC group, *P. copri* treatment and *L. murinus* treatment by themselves notably reduced P-SMAD2, and administration of *P. copri* combined with *L. murinus* led to a remarkable reduction in the levels of SMAD2 and P-SMAD2 (Figure 4C). In addition, as shown in Figure 4D, SMAD3 protein expression was significantly activated by the DDC diet. When *P. copri* and *L. murinus* were used as interventions in the PSC mice alone, the SMAD3 protein level could not be significantly restored; in contrast, when both bacteria were administered together, significant reduction in SMAD3 expression was observed. P-SMAD3 protein was significantly inhibited by the bacterial interventions and could be restored to normal level after *P. copri* and *L. murinus* treatments alone or in combination. Collectively, these results suggest that *P. copri* and *L. murinus* treatments might alleviate DDC-induced liver fibrosis by inhibiting TGF-β1/Smad signaling, and the effect of combining *P. copri* with *L. murinus* is more obvious.

### 2.5. Changes in the Microbiome of Mice in Different Treatment Groups Related to P. copri and L. murinus Interventions and Correlations between Different Bacterial Genera and Indicators

**Figure 5:**
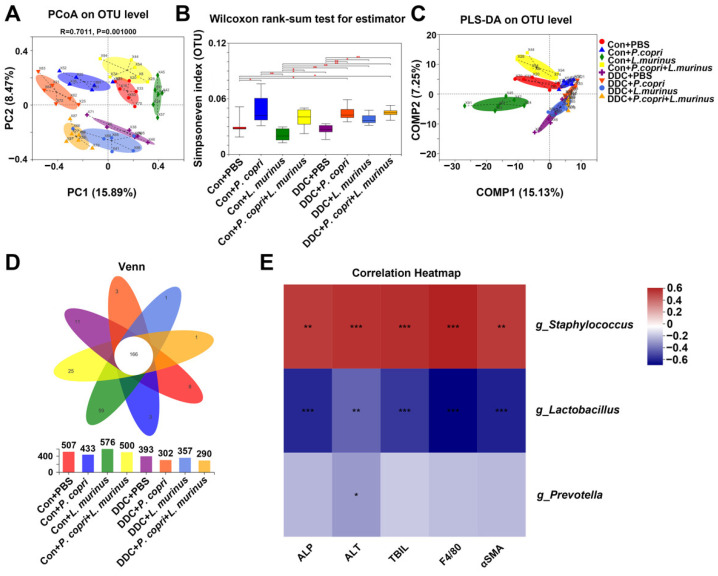
*P. copri* and *L. murinus* affect the structure and diversity of intestinal microbiota and have a significant negative correlation with the observed indicators. (**A**) PCoA analysis was performed on the intestinal microbiota of eight groups of mice at the OTU (Operational Taxonomic Unit) level, and significant differences were found between groups. (**B**) Using Simpsoneven index as the reference, the intestinal microbiota of each group shows different diversity and evenness. (**C**) PLS-DA analysis of classification reliability of each group, showing that the control group and DDC group can be well separated. (**D**) Venn diagram shows the number of different and common OTUs in different treatment groups. (**E**) Two matrices were used to analyze the correlation between the abundance values of *g_Staphylococcus (*“g_” is short for “genus of”), *g_Lactobacillus,* and *g_Prevotella* and the quantitative values of ALP, ALT, TBIL, F4/80, and αSMA, * *p* < 0.05, ** *p* < 0.01, *** *p* < 0.001.

The PCoA analysis demonstrated that each group was distinguishable from others at the OTU level (Figure 5A) and, compared to the intestinal microbiota diversity of the mice in the DDC group, the microbiota diversity was significantly higher in the DDC + *P. copri*, DDC + *L. murinus,* and DDC + *P. copri* + *L. murinus* groups (Figure 5B). The PLS-DA analysis further showed that each of the four control groups could be distinguished from each other, while there was a partial overlap in the background of the DDC treatment groups (Figure 5C), indicating that there are some common genera in the gut microbiota of PSC mice after treatment with *P. copri* and/or *L. murinus*. A Venn diagram was used to analyze the number of common and unique OTUs in the intestinal microbiota of each group of mice, and it was found that the number of OTUs decreased after the DDC diet, with a total of 166 identical out sequences (Figure 5D). Two-matrix correlation analysis was performed between the values of *g_Prevotella*, *g_Lactobacillus,* and *g_Staphylococcus,* which had the most abundant OTU values, and the detected values of ALP, ALT, TBIL, F4/80, and αSMA (Figure 5E). The results showed a significant positive correlation between *g_Staphylococcus* and changes in these indicators (r = 0.44, *p* = 0.002; r = 0.46, *p* = 0.0009; r = 0.47, *p* = 0.0007; r = 0.50, *p* = 0.0003; and r = 0.44, *p* = 0.002, respectively), while *g_Lactobacillus* showed a significant negative correlation (r = −0.56, *p* < 0.001; r = −0.43, *p* = 0.002; r = −0.53, *p* = 0.0001; r = −0.65, *p* < 0.001; and r = −0.58, *p* < 0.001, respectively) and *g_Prevotella* showed only a statistically significant negative correlation with ALT (r = −0.31, *p* = 0.03). The above results illustrated that the increase in *Lactobacillus* abundance was significantly correlated with the recovery of serum biochemical indicators ALP, ALT, and TBIL (Figure 1C), the decrease in macrophage number (Figure 2C), and the decrease in αSMA-positive area indicators (Figure 3D). The addition of *Prevotella* significantly modulated the reduction in ALT indicators in Figure 1C, suggesting the addition of bacteria led to a significant improvement in DDC-induced liver injury, inflammation, and fibrosis.

### 2.6. Inner Communication and Fluorescence Localization of P. copri and L. Murinus in the Intestines of Mice

**Figure 6:**
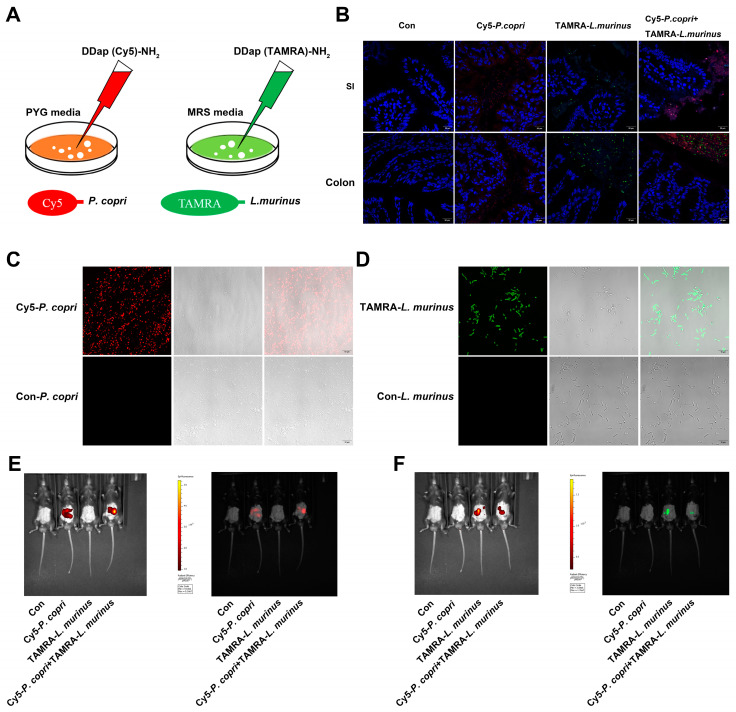
*P. copri* and *L. murinus* were labeled by Cy5 and TAMRA fluorescence, respectively, and intestinal coexistence was observed after gavage. (**A**) *P. copri* and *L. murinus* were cultured in a medium added with 300 μM Cy5 (red fluorescence) and TAMRA (green fluorescence), respectively, and fluorescence-labeled bacteria were obtained after 24 h (**C**,**D**; scale bar = 10 μm). The fluorescence intensity of fluorescence-labeled Cy5-*P. copri* (**E**) and TAMRA-*L. murinus* (**F**) in the intestinal tract was observed via in vivo fluorescence imaging. (**B**) The frozen sections of the small intestine and colon were taken quickly, and the cell nuclei were stained with DAPI to observe the location of fluorescence-labeled bacteria (scale bar = 20 μm).

According to the above results, we suspected that *P. copri* and *L. murinus* might be co-located in the intestine and regulate hepatic fibrosis and inflammation-related signaling pathways through the liver–intestine axis to improve their synergistic effects. We constructed fluorescence-labeled strains of *P. copri* and *L. murinus* named Cy5-*P. copri* and TAMRA-*L. murinus*, in which two FDAA probes, the D-type amino acids DDap (Cy5)-NH_2_ and DDap (5TAMRA)-NH_2_, were added into the media to separately cultivate these strains, as shown in Figure 6A. The fluorescence-labeled bacteria could be clearly observed by changing the excitation wavelength under a laser confocal microscope after in vitro culture (Figure 6C,D). The in vivo imaging observation showed that both green-fluorescence-labeled Cy5-*P. copri* and red-fluorescence-labeled TAMRA-*L. murinus* localized in the gastrointestinal tract in the gavaged mice (Figure 6E,F). As expected, observation of the frozen sections of the SI and colon using confocal microscopy further confirmed that the two types of fluorescence were visible in the same location in the intestine when the two strains were administered together (Figure 6B). These results indicated that *P. copri* and *L. murinus* were stably present in the intestine and could coexist within the same location, providing a theoretical foundation for the combined intervention of *P. copri* and *L. murinus* in PSC mice to improve their liver injury, inflammation, and fibrosis.

## 3. Discussion

It has been reported that a change in gut microbiome composition is not only associated with some diseases but also modulates host metabolism [22,23,24,25]. Our previous study found that *P. copri* intervention significantly improved cholestasis and BA metabolism in PSC mice [9], but *P. copri* failed to reduce liver inflammation. Therefore, we speculated that the combination of beneficial bacteria might improve disease status in PSC mice, including reducing inflammation. Previous research studies reported that *L. murinus* was able to mediate anti-inflammatory effects in calorie-restricted mice [11], and we found that *Lactobacillus* showed a significant decrease in abundance after the DDC diet and an increase in abundance after the DDC + *P. copri* treatment in our earlier studies [9], suggesting that the addition of *L. murinus* might help *P. copri* to exert anti-inflammatory effects in PSC mice. In line with our hypothesis, the combined intervention of *P. copri* and *L. murinus* effectively improved liver injury and inflammatory and fibrotic processes in our DDC-induced PSC mouse model (Figure 2). Moreover, the combined intervention of the two bacteria significantly reduced the expression of inflammatory factors such as *Mpeg1* and *Mcp-1* (Figure 2D). *Mpeg1* is a gene that encodes macrophage-specific expression, which represents changes in the number of macrophages [26,27]. *Mcp-1* is a chemokine that specifically acts on monocytes to accelerate the aggregation of monocytes [28,29]. It has been found that activation of monocytes and macrophages is stimulated by the microenvironment in vivo, including infections by pathogens such as *Mycobacterium tuberculosis* infection, delayed hypersensitivity reactions and other diseases [30,31]. This leads to some qualitative and quantitative changes in the macromolecular components of the macrophage membrane and promotes inflammation [32,33]. In our previous study, we discovered that only when the intestinal mucosa was damaged by treatment with the DDC diet did *P. copri* breach the barrier into the internal environment to induce inflammation; in contrast, when the integrity of the gut barrier was preserved, this pro-inflammatory effect of *P. copri* did not manifest [9]. Therefore, the DDC diet induced severe liver inflammation and fibrosis in the PSC mice by recruiting a large number of macrophages and monocytes. The combined intervention of *P. copri* and *L. murinus* significantly reduced the response of macrophages and monocytes in the PSC mice, and the improvement in inflammatory immune response in the PSC mice was characterized by the expression of *Mpeg1* and *Vacm-1*.

Previous research has reported that *Staphylococcus* is the most common purulent cocci, and organisms are susceptible to its infection when their skin mucosa is traumatized or when the immunity of the organisms is reduced [34,35]. In our study, the 16S rRNA intestinal microbiota sequencing analysis indicated that *g_Staphylococcus* was the most abundant genus and showed a significant positive correlation with the expression of ALP, ALT, TBIL, F4/80, and αSMA, suggesting that its increased abundance contributed to the exacerbation of disease indications in the DDC-induced PSC mice (Figure 5E). Unlike *Staphylococcus*, *Prevotella* and *Lactobacillus* were negatively correlated with the expression of ALP, ALT, TBIL, F4/80, and αSMA, suggesting that an increase in *P. copri* and *L. murinus* abundance after the addition of the bacterial intervention contributed to reducing disease indicators in the PSC mice. This difference might be due to the enterotoxin produced by *Staphylococcus* during proliferation, which harms the metabolism of the gut microbiota and leads to disease symptoms in vivo [36]. Both *P. copri* and *L. murinus* have been found to regulate metabolism in vivo; for example, *P. copri* can help decompose proteins and carbohydrates [37,38], and *L. murinus* can produce lactic acid through fermentation of carbohydrates, promote animal growth, and regulate normal gastrointestinal microbiota [10,39]. Other studies have shown that *Lactobacillus* can significantly activate the phagocytosis of macrophages, stimulate peritoneal macrophages, and induce the production of interferon, which have special advantages in improving inflammation and fibrosis [40,41,42,43]. This is consistent with our findings, as shown in Figure 2, that *L. murinus* significantly reduced liver inflammation in the PSC mice after intervention. However, variations in the abundance of *Staphylococcus*, *Prevotella,* and *Lactobacillus* genera found in this study did not have a direct effect on liver inflammation and fibrosis; rather, the effect was exerted through an intermediary, which warrants further investigation.

TGF-β1 and the SMAD protein family are pivotal players in the development and progression of liver fibrosis [16,44,45,46,47]. In hepatic stellate cells, TGF-β1 produced by paracrine and autocrine can strongly stimulate the synthesis of collagen type Ⅰ and other mechanistic components [48,49]. It has been confirmed that TGF-β1 can encode the expression of major extracellular matrix component genes and regulates the progression of liver fibrosis by activating metalloproteinase inhibitors to inhibit metalloproteinase activity [50,51]. SMAD-family proteins play a key role in the transmission of TGF-β1 signals from cell surface receptors to the nucleus, and different SMADs mediate the signal transduction of different TGF family members [52,53,54]. According to their functions, SMAD proteins can be divided into three subfamilies: receptor-activated SMAD (R-SMAD), common pathway SMAD (Co-SMAD), and suppressed SMAD (I-SMAD) [44,55]. R-SMAD can be activated by TGF-β1 receptors and form complexes with them, including SMAD2 and SMAD3. It has been pointed out that with the aggravation of liver fibrosis, the expression of SMAD2 and SMAD3 increases [56]. In a previous study, SMAD3 knockout mice had a lower degree of liver fibrosis induction than wild-type mice [57]. Co-SMAD, including SMAD4, is a medium commonly required in the signaling process of the TGF family. Studies have found that increasing the content of SMAD4 can reduce the synthesis of hepatic ECM, thus slowing down the process of liver fibrosis, which has potential clinical application value [58]. I-SMAD includes SMAD6 and SMAD7, which bind to the TGF complex and inhibit or regulate TGF-β1 signaling to improve liver inflammation and fibrosis [59]. The DDC-induced PSC mouse model used in this study presented severe liver inflammation and fibrosis. By detecting the expression levels of TGF-β1 and SMAD proteins in vivo (Figure 4), it was found that DDC activated the TGF-β1/Smad signaling pathway and significantly increased the expression of TGF-β1 and SMAD 2/3 proteins to induce liver inflammation and fibrosis. After being treated with the *P. copri* and *L. murinus* combined intervention, the reception and binding of TGF-β1 signal decreased, and the relative level of SMAD 2/3 also significantly decreased, suggesting that the TGF-β1/Smad signaling pathway might be inhibited by the bacterial intervention, thus leading to significant improvement in liver inflammation and fibrosis in the PSC mice. Furthermore, PSC is more common in men, although there is also a high number of women with PSC [2,60]. Female sex and estrogens are important regulators of bile acid production through critical hepatic feedback mechanisms, affecting enzyme activity and bile acid pool composition [61]. Moreover, we have confirmed that *P. copri* treatment regulates the metabolism of bile acids; thus, the interference caused by estrogen in the experiment was substantially attenuated and we chose male mice in this study [9]. We believe that our pioneering work has validity and will further explore the gender factor in future studies.

## 4. Materials and Methods

### 4.1. Culture and Quantification of Bacterial Strains

*P. copri* (DSM18205), blood agar base, and sterile defibrinated sheep whole blood were acquired from Mingzhou Biotechnology Co., Ltd. (Ningbo, China). Both *L. murinus* (BNCC194688) and MRS medium (lactic acid bacterial culture medium) were purchased from BeNa Culture Collection (Beijing, China). The above bacteria were cultured in an anaerobic culture tank (volume: 2.5 L, Mitsubishi, Tokyo, Japan) equipped with anaerobic gas-producing bags and indicators to provide an anaerobic environment. Colonies were cultured using Gram stain to determine the bacterial species. Bacterial DNA was extracted using the TIANamp Bacteria DNA Kit (Tiangen Biotechnology Co., Ltd., Beijing, China). qPCR was performed using a QuantStudio^®^ 5 Real-Time System (Applied Biosystems, Carlsbad, CA, USA), and the cyclic conditions were as follows: 95 °C for 5 min, 40 cycles at 95 °C for 10 s, 56 °C for 20 s, and 72 °C for 30 s. *Eub* was used as an internal reference to calculate the relative expressions using the 2^−ΔΔCt^ calculation method. The primer sequences used are shown in Table 1.

### 4.2. Animal Experiments

Male 7-week-old C57BL/6J specific pathogen-free (SPF) mice were purchased from GemPharmatech Co., Ltd. (Nanjing, China) and housed in a 12 h light/dark cycle, with no restrictions on their food or water under conditions of controlled humidity (50 ± 5%) and temperature (22 ± 2 °C). All experiments involving mice were approved by the Nanjing Medical University Institutional Animal Care and Use Committee (Approval No. IACUC-2005013). A total of 80 mice were randomly divided into 8 groups of 10 mice each for the experimental treatment, and there was no significant difference in mouse body weight between the 8 groups. All experiments involving mice were conducted in parallel. There was a three-day adaptation period after the mice entered the facility. All mice in the facility drank purified water after disinfection. The mice were randomly divided into eight groups using random numbers based on the weight of each mouse, and statistical analysis of the body weight of the mice in each group after grouping showed no significant differences. The eight groups of mice were divided into four groups each of Con (short for control) and DDC groups and fed a normal or a customized diet for one week, respectively. The customized diet was 0.1% (*w*/*w*) DDC (CAS: 632-93-9; Sigma-Aldrich Co., Ltd., Saint Louis, MO, USA) supplemented diet synthesized by Jiangsu Xietong Pharmaceutical Bio-engineering Co., Ltd. (Nanjing, China). The mice in the diet model group were raised separately from the other groups to ensure they maintained their SPF status. After that, the eight groups of mice were treated with gavage for one week with continuous feed supply, with four groups each of control and DDC groups, and each group was gavaged with the same volume of PBS (0.01 M, pH 7.2–7.4), *P. copri*, *L. murinus*, and a mixture of the two bacterial suspensions. The bacterial suspensions were washed, centrifuged, and re-suspended in PBS. A microplate reader read that OD_600nm_ was equal to 1, which corresponds approximately to a strain concentration of 1 × 10^9^ CFU/mL. All mice were sacrificed after 12 h of starvation on the last day, and fresh small intestinal contents were collected. The collected blood was stored overnight at 4 °C, and the serum was collected via centrifugation. Liver tissues were immediately excised, sectioned, and fixed for further histological and immunohistochemical analysis, with the remainder stored at −80 °C until use.

### 4.3. Analysis of Serum Biochemical Indicators

Serum biochemical indicators, including alanine aminotransferase (ALT), alkaline phosphatase (ALP), and total bilirubin (TBIL), were detected using an automatic biochemical analyzer (Hitachi 7100, Hitachi, Ltd., Tokyo, Japan).

### 4.4. Determination of Total Bile Acids (TBAs)

The content of TBAs in the liver and intestine tissues were determined using TBA kits (No. E003-2-1; Jiancheng Bioengineering Institute, Nanjing, China).

### 4.5. Histopathology

The liver tissues were fixed in 4% paraformaldehyde for over 24 h, paraffin-embedded, cut to 4 μm thick, and stained with hematoxylin and eosin (H&E), Sirius red (SR) and immunohistochemical (IHC) staining. The IHC staining was used to analyze epithelial cell proliferation (cytokeratin 19, CK19), immune cell infiltration (mouse EGF-like module-containing mucin-like hormone receptor-like 1, EMR1, F4/80), and liver fibrosis (α-smooth muscle actin, αSMA) in the bile duct. We performed IHC staining with the following primary antibodies: (a) rabbit anti-αSMA (Servicebio, Wuhan, China, GB111364); (b) rabbit anti-CK19 (Servicebio, GB11197); and (c) rabbit anti-F4/80 (Servicebio, GB113373). Standard procedures of IHC staining were used as described in a previous study [9]. Representative images were randomly selected and recorded by specialized pathologists without knowledge of the group assignment in a blinded manner using Panoramic scanning (3DHISTECH, Budapest, Hungary). The images were then analyzed and quantified using ImageJ.

### 4.6. RNA Extraction and Quantitative Real-Time PCR

Total RNA was extracted from the liver tissues using the FastPure Cell/Tissue Total RNA Isolation Kit (Cat: RC101-01; Vazyme Biotech Co., Ltd., Nanjing, China) and reverse transcribed to cDNA using the HiScript II Q RT SuperMix (Cat: R223-01; Vazyme Biotech Co., Ltd.). Total RNA was measured for concentration and purity using a NanoDrop2000 spectrophotometer (NanoDrop Technologies; Thermo Fisher Scientific, Inc., Waltham, MA, USA). Then, real-time PCR was used to quantitatively detect the gene expression. Enriched cDNA, forward primer and reverse primer (0.2 μM, respectively, see Table 1 of oligonucleotides sequences; synthesized by Genscript Biotech Corporation, Nanjing, China), and Hieff^®^ qPCR SYBR^®^ Green Master Mix (5 μL, Cat: 11202ES08, Yeasen Biotech Co., Ltd., Shanghai, China) were mixed to a volume of 10 μL. The mixture was subjected to qPCR on AB RT-PCR (Q5). *Gapdh* was used as an internal control for target gene expression levels, and the relative gene expression levels were assessed using the 2^−ΔΔCt^ method.

### 4.7. Extraction of Tissue Protein and Western Blot Analysis

Extraction of total protein from the liver tissues was performed using RIPA lysis buffer (Beyotime Biotechnology, Shanghai, China) with protease inhibitor and homogenized using Tissue Lyser II (Dusseldorf, Germany). Protein concentrations were determined using Pierce BCA protein assay reagent (Beyotime Biotechnology, Shanghai, China). After boiling with 1× loading buffer, proteins were separated via electrophoresis on 12% SDS-PAGE gels and subsequently transferred to polyvinylidene fluoride (PVDF) membranes (Millipore, Billerica, MA, USA). Then, the membranes were incubated overnight at 4 °C with the following primary antibodies: (a) rabbit anti-αSMA (1:5000, Servicebio, GB111364); (b) mouse anti-GAPDH (1:10,000, Immunoway, Plano, USA, YM3029); (c) rabbit anti-SMAD2 (1:1000, AiFang, Changsha, China, AF03498); (d) rabbit anti-P-SMAD2 (1:5000, AiFang, AF00740); (e) rabbit anti-SMAD3 (1:5000, AiFang, AF03501); and (f) rabbit anti-P-SMAD3 (1:5000, AiFang, AF00904). Afterwards, the membranes were incubated with a secondary antibody for 2 h at room temperature, and the bands were visualized using Chemiluminescent HRP Substrate (Millipore Corporation, Billerica, MA, USA). All experiments were repeated at least three times. The results were normalized to the GAPDH correspondence and analyzed using ImageJ software (NIH, Bethesda, MD, USA).

### 4.8. 16S rRNA Sequencing

Microbial DNA was extracted from the small intestine samples using the E.Z.N.A.^®^ soil DNA Kit (Omega Bio-tek, Norcross, GA, USA) according to the manufacturer’s protocols. The final DNA concentration and purification were determined using a NanoDrop 2000 UV-vis spectrophotometer (Thermo Scientific, Wilmington, NC, USA), and DNA quality was checked using 1% agarose gel electrophoresis. The V3-V4 hypervariable regions of the bacterial 16S rRNA gene were amplified with primers 338F and 806R using a thermocycler PCR system (GeneAmp 9700, ABI, Carlsbad, CA, USA). Sequencing was pooled on an Illumina MiSeq platform. The classification of each 16S rRNA gene sequence was analyzed using the RDP Classifier algorithm (http://rdp.cme.msu.edu/, accessed on 15 July 2021) against the Silva 138/16S rRNA database (https://www.arb-silva.de/, accessed on 15 July 2021) with confidence threshold of 70%. The data were stored and analyzed using the I-Sanger Bio-cloud platform by Shanghai Majorbio Bio-pharm Technology Co., Ltd. (Shanghai, China).

### 4.9. Bacterial Labeling with Fluorescent Probes

Two fluorescent D-amino acid (FDAA) probes were purchased from Chinese Peptide Company (Hangzhou, China): (a) DDap (Cy5)-NH_2_, with 95% purity, excitation light wavelength (EX) = 649 nm, and emitted light wavelength (EM) = 670 nm, and (b) DDap (5TAMRA)-NH_2_, with 95% purity, EX = 555 nm, and EM = 580 nm. The structure and dosage were used as described in [62]. *P. copri* and *L. murinus* were cultured to an early stationary phase. FDAA was then added to the culture media to a final concentration of 300 μM. The DDap (Cy5)-NH_2_ marker for *P. copri* and DDap (5TAMRA)-NH_2_ marker for *L. murinus* were used, respectively. Two hours after the bacteria were labeled, they were washed twice with PBS and re-suspended in PBS to achieve an appropriate concentration for analysis using the Small Animal Live Visible 3D Imaging System (IVIS Spectrum, PerkinElmer, Waltham, MA, USA). Cy5-*P. copri* and 5TAMRA-*L. murinus* were administered by gavage to different groups of mice in a volume of 200 µL each, and another group of mice was gavaged with an equal amount of PBS as a reference. Immediately after the in vivo visible light 3D imaging of the mice, each intestinal segment was removed, frozen sections were created, the nucleus was stained with DAPI, and the location of bacteria in each intestinal segment was observed under a confocal microscope (Zeiss LSM700, Oberkochen, Germany).

### 4.10. Statistical Analysis

The data in this study were expressed as mean ± standard deviation (SD). Statistical comparisons were analyzed using SPSS (Version 20). For data that conform to the normality, homogeneity of variance was tested using Levene’s test, and then a t-test was carried out to calculate significant differences between groups. A Kruskal–Wallis test was used when data did not follow a normal distribution. Bonferroni correction was used to adjust the overall significance level. The data were plotted using GraphPad Prism 8 (GraphPad, La Jolla, CA, USA). Special and specific analysis was presented in the legend of each corresponding chart. *p* < 0.05 was considered statistically significant.

## Figures and Tables

**Table 1:** List of primer sequences used in this study.

Gene	Gene Name	Sequence
*Mpeg1*-F	Macrophage-expressed gene 1	CGAAGATGGCCACCTACCTGGCAGA
*Mpeg1*-R		GAAGGCAATCCCTGCAGAAGCGGTC
*Mcp-1*-F	Monocyte chemoattractant protein-1	GATGCAGTTAACGCCCCACT
*Mcp-1*-R		CCCATTCCTTCTTGGGGTCA
*Vcam-1*-F	Vascular cell adhesion molecule-1	GCTCTGTGGGTTTTGAGGATGA
*Vacm-1*-R		GGATCTTCAGGGAATGAGTAGACC
*Cdh1*-F	Cadherin 1	CGTCCTGCCAATCCTGATGA
*Cdh1*-R		ACCACTGCCCTCGTAATCGAAC
*Collagen 1a1*-F	Collagen type I alpha 1	GAATGCAATGAAGAACTGGACTGT
*Collagen 1a1*-R		TCCTACATCTTCTGAGTTTGGTGA
*Timp-1*-F	Tissue inhibitor of metalloproteinase 1	CATGGAAAGCCTCTGTGGAT
*Timp-1*-R		AAGCTGCAGGCATTGATGTG
*Mmp9*-F	Matrix metalloproteinase 9	CGCCTTGGTGTAGCACAACA
*Mmp9*-R		ACAGGGTTTGCCTTCTCCGTT
*Tgf-β1*-F	Transforming growth factor-beta 1	TCAGACATTCGGGAAGCAGT
*Tgf-β1*-R		ACGCCAGGAATTGTTGCTAT
*Tgf-βrⅡ*-F	Transforming growth factor-beta receptor Ⅱ	GGCTCTGGTACTCTGGGAAA
*Tgf-βrⅡ*-R		AATGGGGGCTCGTAATCCT
*Tgf-βrⅠ*-F	Transforming growth factor-beta receptor Ⅰ	GGCGAAGGCATTACAGTGTT
*Tgf-βrⅠ*-R		TGCACATACAAATGGCCTGT
*Smad2*-F	Mothers against decapentaplegic homolog 2	TGTAGACGGCTTCACAGACC
*Smad2*-R		TCACTTAGGCACTCAGCAAAC
*P. copri*-F	*Prevotella copri*	CCGGACTCCTGCCTGCAA
*P. copri*-R		GTTGCGCCAGGCACTGCGAT
*L. murinus*-F	*Lactobacillus murinus*	TAGGATTGTCAAAAGATGTC
*L. murinus*-R		AGCTAGTTGGTGGGGTAAAG
*Eub*-F	The universal primer sequence of 16S for bacteria	AGAGTTTGATCCTGGCTC
*Eub*-R		TGCTGCCTCCCGTAGGAGT
338F	Bacterial-targeting universal primers (MiSeq sequencing platform)	ACTCCTACGGGAGGCAGCAG
806R		GGACTACHVGGGTWTCTAAT

## Data Availability

The data presented in this study are available from the corresponding author upon request. The data are not publicly available as they will be utilized for future research studies.

## Supplementary Materials

The following supporting information can be downloaded at: https://www.mdpi.com/article/10.3390/ijms241311010/s1.
