# Supplementary material for: Combined Inhibition of the TGF-β1/Smad Pathway by Prevotella copri and Lactobacillus murinus to Reduce Inflammation and Fibrosis in Primary Sclerosing Cholangitis
Source: Int J Mol Sci. 2023 Jul 2;24(13):11010. doi: 10.3390/ijms241311010 (PMC10341692; doi:10.3390/ijms241311010)
Supplement: Supplementary file 1 [file ijms-24-11010-s001.zip › ijms-2436203-supplementary.pdf]

Supplement

Figure S1 Identification of *Prevotella copri* (*P. copri*) and *Lactobacillus murinus* (*L. murinus*).

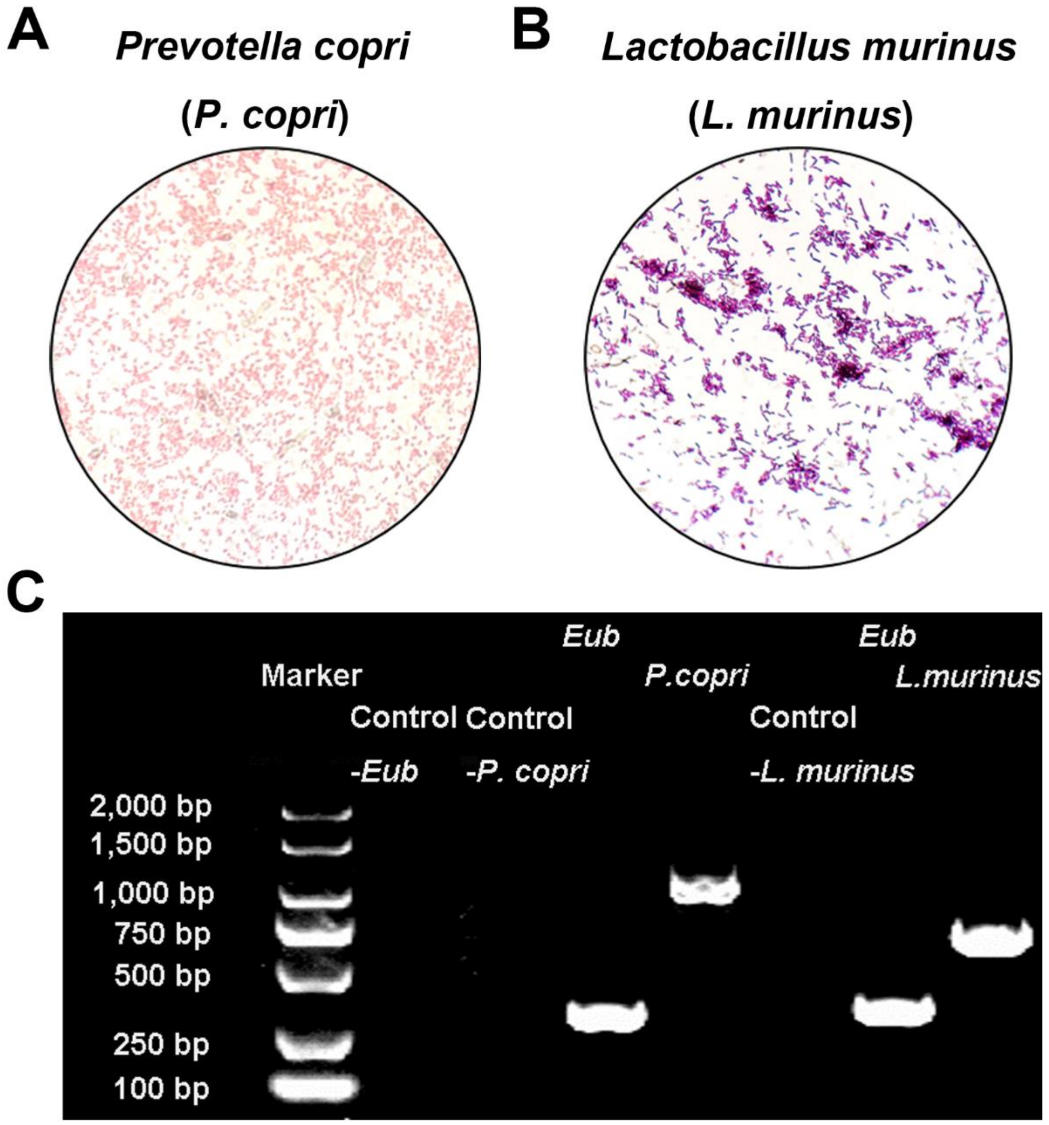

The *P. copri* and *L. murinus* were validated by two methods. (A) Under oil microscope, *P. copri* was gram-negative and *L. murinus* was gram-positive. (B) The specific primers of *P. copri*, *L. murinus* and the universal primers EUB were used for PCR verification, and the bands were visible by 2% agarose gel electrophoresis.

We perform IHC with the following primary antibodies:

(a) rabbit anti-αSMA (alpha smooth muscle Actin rabbit polyclonal antibody, Servicebio, GB111364);

(b) rabbit anti-CK19 (Cytokeratin 19 rabbit polyclonal, Servicebio, GB11197);

(c) rabbit anti-F4/80 (F4/80 rabbit polyclonal, Servicebio, GB113373).

According to standard procedures, the main experimental steps included fixing, paraffinizing, deparaffinizing, dehydrating, antigen retrieval, blocking endogenous peroxidase activity, sealing, primary antibody incubation, washing, sealing, secondary antibody incubation, washing, chromogenic, nucleus counterstaining, dehydration and mounting, and observation.
